# Surgical and Conservative Treatment of Post-infarction Left Ventricular Pseudoaneurysm

**DOI:** 10.3389/fcvm.2022.801511

**Published:** 2022-01-27

**Authors:** Zhaoji Zhong, Wu Song, Shanshan Zheng, Sheng Liu

**Affiliations:** Department of Cardiovascular Surgery, Fuwai Hospital, Chinese Academy of Medical Science, Beijing, China

**Keywords:** left ventricle, pseudoaneurysm, false aneurysm, myocardial infarction, surgery

## Abstract

**Background:**

Post-infarction left ventricular (LV) pseudoaneurysm is a rare mechanical complication of myocardial infarction that carries a substantial risk of sudden rupture. The purpose of this study was to compare the surgical results of post-infarction LV pseudoaneurysm with those of conservative treatment.

**Methods:**

From 2016 to 2021, 22 patients were hospitalized for LV pseudoaneurysm, including 17 cases (77.3%) caused by myocardial infarction. Of the 17 patients, 10 (58.8%) underwent surgical repair, while seven (41.2%) were treated medically. The clinical course, echocardiograph data, and surgical outcomes were analyzed. Survival rates of the surgical and conservative groups were compared.

**Results:**

There were no perioperative deaths. Intra-aortic balloon pumping support was required in two (20%) patients. No follow-up mortality was observed in the surgical group and at the last follow-up, all the patients were classified as New York Heart Association class I–II. In the conservative group, there was one (14.3%) hospital death and two (28.6%) additional deaths during follow-up. A significant difference was found in survival between the two groups (*P* = 0.024).

**Conclusions:**

Surgical repair of post-infarction LV pseudoaneurysm can be performed with good results, while conservative treatment carries a significant risk of sudden death. Surgical repair is indicated for every patient diagnosed, even those with a small pseudoaneurysm without symptoms.

## Introduction

Left ventricular (LV) pseudoaneurysm, or false aneurysm, develops when cardiac rupture is small or “oozing,” and allows adhesion formation between the epicardium and pericardium (1). Although it may occur after valve surgery, infective endocarditis, or chest trauma, the most common etiology is acute myocardial infarction (AMI), of which it is a rare mechanical complication (2).

In the era of percutaneous coronary intervention (PCI) and mechanical circulatory support (MCS), the rate of mechanical complications is low, but mortality and morbidity remain high (3, 4). Surgical and percutaneous repair of LV pseudoaneurysms have been reported (5), and small series and case reports have shown optimistic surgical results (6). However, because of its rarity, its natural history and optimal treatment have not been established (2, 7, 8).

Thus, the aims of this study were to: (1) determine the operative and late results of post-infarction LV pseudoaneurysm, and (2) compare the results with conservative treatment.

## Patients and Methods

From 2016 to 2021, 22 consecutive patients were hospitalized for LV pseudoaneurysms. The etiologies included myocardial infarction in 17 cases (77.3%), valvular surgery in three cases (13.6%), infective endocarditis in one case (4.5%), and in one case (4.5%) it was undefined. Patients with causes other than myocardial infarction or true aneurysms were excluded from this study. Data of the 17 cases of post-infarction LV pseudoaneurysm were obtained retrospectively and followed-up. This study was approved by the institutional ethics review board of the Fuwai Hospital.

All patients had a history of AMI. Seventeen patients (100%) were diagnosed with LV pseudoaneurysm by transthoracic echocardiography, which was confirmed by magnetic resonance imaging (MRI) in nine (52.9%) and computed tomography angiography (CTA) in 10 patients (58.8%). The diagnosis was confirmed by intraoperative findings in all patients who underwent surgery. The wall consisted of mural thrombi and the pericardium alone. In 9 patients (90%), some aneurysmal wall and adherent pericardium were resected, and the resected tissue was examined pathologically, which validated the diagnosis.

Patient baseline characteristics are shown in Table 1. The most common location of the pseudoaneurysm was the posterior-inferior wall (*n* = 10, 58.8%), followed by the lateral wall (*n* = 5, 29.4%), and anterior wall (*n* = 2, 11.8%). The AMI-hospitalization interval was 4.4 (1.3~13.8) months for the surgical group and 5.7 (1.6~36.5) months for the conservative group. In the surgical group, two (20%) patients underwent surgery <1 month after AMI, and four (40%) > 3 months after AMI. The interval between pseudoaneurysm diagnosis and surgery was 15.3 ± 9.1 days (range, 4–29 days). There was no significant difference in the baseline characteristics between the two groups (Table 2).

**Table 1:** Clinical characteristics of patients with LV pseudoaneurysm.

**No**.	**Gender/age**	**Main presentation**	**Euroscore II**	**Location LVEF**	**Coronary disease**	**PA diameter (mm)**	**Neck diameter (mm)**	**Repair technique**	**Associate procedures**	**Peri-operative morbidity**	**Follow-up (months)**
**Surgical group**											
1	M/48	CHF	9.01%	Posterior, 40%	1-vessel Post-PCI	54	40	Patch	No		Survivor, 176^*^
2	M/59	CHF	4.98%	Lateral, 40%	2-vessels	98	29	Patch	CABG*1+MVR		Survivor, 63
3	M/51	CHF	3.13%	Lateral, 39%	2-vessels	68	50	Patch	CABG*1		Survivor, 47
4	M/57	CHF	3.62%	Lateral, 32%	2-vessels Post-PCI	86	49	Patch	CABG*2 +AVR	IABP	Survivor, 32
5	F/72	Angina	10.13%	Inferior, 56%	2-vessels	64	13	Direct suture	No		Survivor, 33
6	F/62	Angina	3.28%	Inferior, 60%	3-vessels	35	4	Direct suture	CABG*2	Sternal wound dehiscence	Survivor, 4.7
7	M/74	CHF	7.86%	Posterior, 57%	1-vessel	29	5	Direct suture	CABG*1+MVR		Survivor, 1.8
8	M/58	Angina	3.09%	Inferior, 47%	3-vessels	20	4	Direct suture	CABG*3		Survivor, 4.7
9	M/51	CHF	7.86%	Anterior, 35%	2-vessels	55	13	Direct suture	CABG*2	IABP	Survivor, 2.4
10	M/69	Angina	7.35%	Lateral, 50%	3-vessels	28	9	Direct suture	CABG*2		Survivor, 2.0
**Conservative group**											
1	M/69	CHF	10.17%	Anterior, 36%	1-vessel	9	3				Death, 24
2	M/53	Asymptomatic	3.91%	Inferior, 60%	Post-CABG	103	4				Survivor, 32
3	M/76	Angina	3.21%	Lateral, 54%	2-vessels Post-PCI	8	3				Death, 0.3 M
4	M/46	Asymptomatic	1.53%	Posterior, 60%	2-vessels	115	56				Death,0.7 M
5	M/73	Asymptomatic	2.55%	Inferior, 58%	3-vessels	42	14				Survivor, 8.1 M
6	M/60	Angina	4.68%	Inferior, 40%	3-vessels Post-PCI	20	2				Survivor, 4.9 M
7	M/65	Angina	3.43%	Posterior, 55%	1-vessel	43	12				Survivor, 2.1 M

* *The patient was hospitalized for non-cardiac causes during the study period and was included in the study, with the LV pseudoaneurysm repaired in 2007*.

*PA, pseudoaneurysm; LVEF, left ventricular ejection fraction; CHF, congested heart failure; CABG, coronary artery bypass grafting; MVR, mitral valve repair; AVR, aortic valve repair; IABP, intra-aortic balloon pumping; PCI, percutaneous coronary intervention*.

**Table 2:** Comparison of baseline characteristics between the two groups.

	**Surgical (*n* = 10)**	**Conservative (*n* = 7)**	**P**
Age, year	60.1 ± 9.1	63.1 ± 10.9	0.540
Female	2 (20%)	0	1.000
NYHA III~IV	1 (10%)	0	1.000
AMI interval, months	4.4 (1.3~13.8)	23.5 (2.0~209.4)	0.270
Preoperative TTE			
LVEF, %	45.6 ± 9.8	51.9 ± 9.8	0.216
LVEDD, mm	55.0 ± 8.4	53.3 ± 9.5	0.701
Neck, mm	13.0 (4.5~34.5)	8.0 (2.8~24.5)	0.407
Length, mm	53.7 ± 26.0	55.2 ± 43.9	0.934
Width, mm	46.8 ± 26.8	37.7 ± 28.0	0.527
≥Moderate MR	0	1 (10%)	0.412
≥Moderate PE	2 (20%)	0	0.485
Follow-up, months	18.5 (2.2~51.2)	8.1 (0.7~24.0)	0.435

*NYHA, New York Heart Association; TTE, transthoracic echocardiography; LVEF, left ventricular ejection fraction; LVEDD, left ventricular end-diastolic dimension; MR, mitral regurgitation; PE, pericardial effusion*.

Conservative management included maintenance of fluid infusion and inotropic support, as needed. Prolonged bed rest and strict blood pressure control were included in conservative management to prevent rupture. Routine medical therapies for coronary artery disease were initiated in patients without contraindications, including dual-antiplatelet therapy, beta-blockers, nitrates, and statins. For patients with reduced LV function, diuretics were also administered.

Surgery is indicated for every patient with an LV pseudoaneurysm. Once the diagnosis of LV pseudoaneurysm was made, surgery was strongly recommended by our heart team, even for those without symptoms. Seven (41.2%) patients refused surgery after they were informed of the benefits and risks of the surgery. These patients were treated conservatively.

### Surgical Technique

Median sternotomy with hypothermic cardiopulmonary bypass (CPB) was performed in all patients and the ascending aorta was the site of arterial cannulization. Right atrial cannulation with double-stage venous drainage was used in eight (80%) patients, and bicaval cannulation was performed in two (20%) patients who required mitral valve repair.

**Figure 1:**
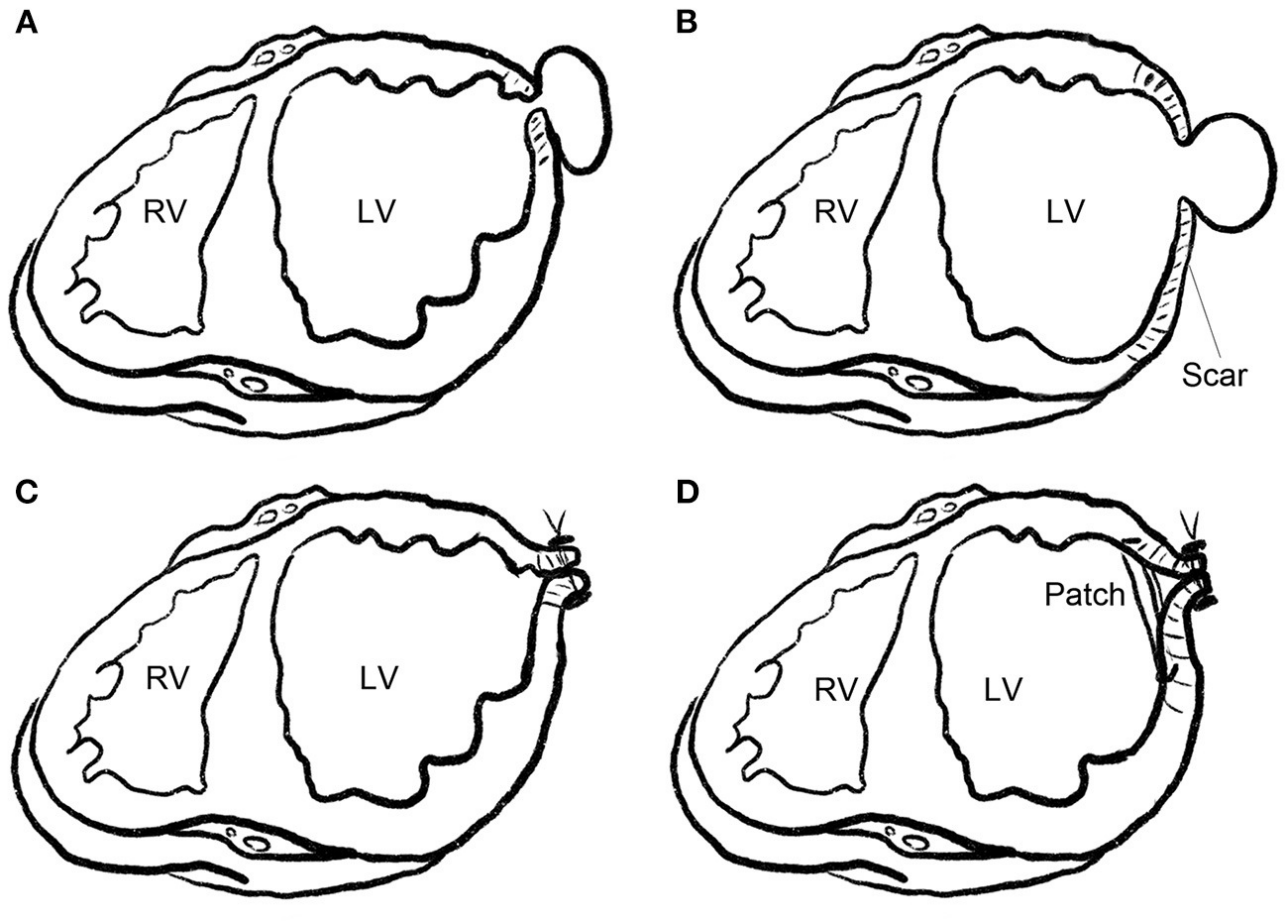
Repair technique for LV pseudoaneurysm. **(A)** Pseudoaneurysm with a narrow neck. **(B)** Pseudoaneurysm with a wide neck or extensive scarring around the neck (pseudoaneurysm superimposed on a true aneurysm). **(C)** Pseudoaneurysm with a narrow neck, as shown in **(A)**, was closed linearly with continuous or horizontal mattress sutures with pledgets. **(D)** Pseudoaneurysm with a wide neck and extensive scaring around the neck, as shown in **(B)**, was closed with a patch, and the scarred wall was trimmed and sutured over the patch.

Severe pericardial adhesions were observed in all the patients. The fibrous tissue around the neck of the pseudoaneurysm was mobilized with care or left adherent to the pericardium. Two different approaches were used to repair the pseudoaneurysm. These techniques were similar to those previously described for true aneurysms (9). The techniques were chosen according to the width of the pseudoaneurysm neck and the adherent tissues. In six (60%) patients with a narrow pseudoaneurysm neck and densely fibrotic edge, the neck was closed linearly with continuous or horizontal mattress sutures with pledgets. In 4 (40%) patients with a wide pseudoaneurysm neck or an extensive scarring around the neck, a patch was required to maintain LV geometry. After repair of the neck, excessive scarred myocardium and adherent pericardium were trimmed and sutured over the patch (Figure 1).

The concomitant procedures and number of grafts are listed in Table 1. Eight (80%) patients had concomitant coronary artery bypass grafts (CABG), and four (40%) had at least one arterial graft. Mitral valve repair was performed in two (20%) patients, and the technique included mitral annuloplasty with a 29-mm prosthetic ring in one case, and commissural closure in the other case.

Medications similar to those in the conservative group, including dual-antiplatelet therapy, beta-blockers, nitrates, and statins, were also initiated postoperatively and continued after hospital discharge.

### Follow-Up

All patients underwent transthoracic echocardiography (TTE) at hospital discharge. Follow-up was performed through outpatient appointments or telephone interviews. Follow-up data were obtained for all patients. The median follow-up duration was 8.1 (2.1–32.5) months for the conservative group, and 18.5 (2.2–51.2) months for the surgical group.

### Statistical Analysis

Continuous data are presented as the mean ± standard deviation for normally distributed parameters, or median (25th−75th percentiles) for non-normally distributed parameters. Categorical variables were presented as frequencies (percentages). The Kolmogorov–Smirnov test was used to test for normality. For continuous, normally distributed data, comparisons between groups were performed using the *t*-test for paired or unpaired samples. For continuous, not normally distributed data, the Mann–Whitney *U*-test was used for independent samples. Comparisons of categorical variables were performed using Fisher's exact test and ordinal variables were compared using the Mann–Whitney *U*-test for independent samples. Survival was evaluated using Kaplan–Meier analysis and for actuarial estimates, the data are presented as the mean ± standard error. Calculations were performed using SPSS version 22 (SPSS Inc., Chicago, IL, USA).

## Results

### Perioperative Outcomes

The CPB time was 142.4 ± 24.0 min, and aortic clamp time were 93.0 (88.0–101.0) min. There were no hospital deaths or major postoperative complications such as re-exploration for bleeding, stroke, acute kidney injury requiring dialysis, or prolonged mechanical ventilation requiring tracheostomy. Intra-aortic balloon pumping (IABP) support was required in two (20%) patients. It was required for one patient while weaning off CPB intraoperatively, and the other was required on the 2^nd^ postoperative day for ventricular arrhythmia. Both patients recovered, and there were no IABP-related complications. There was one case (10%) of superficial sternal wound infection.

The median mechanical ventilation time was 23.5 (15.0–53.0) hours. Mean intensive care unit and postoperative hospital stays were 5.7 ± 4.5 days and 11.0 ± 5.0 days, respectively.

All patients in the surgical group underwent TTE at hospital discharge. The mean left ventricular ejection fraction (LVEF) and mean left ventricular end-diastolic dimension (LVEDD) was 52.5 ± 6.5 % and 49.3 ± 6.5 mm respectively which showed a significant improvement after surgery (*P* = 0.003 and 0.021, respectively). Residual communication at the pseudoaneurysm neck was observed in one (10%) patient who underwent linear closure with two horizontal mattress (“U” shape) sutures with pledgets. A slow flow with width of 1 mm was detected using echocardiography, which resolved during follow-up. No residual mitral regurgitation was found in the two patients who underwent mitral valve repair.

### Follow-Up Outcomes

In the surgical group (*n* = 10), no deaths were reported during the follow-up period. No reoperation or repeat revascularization was required and, at the last follow-up, all patients were New York Heart Association (NYHA) class I–II.

In the conservative treatment group (*n* = 7), there was one (14.3%) hospital death. The patient was a 76-year-old man who died from a pseudoaneurysm rupture seven days after AMI. Two patients (28.6%) died during the follow-up period. Both deaths were cardiac-related. One patient was a 69-year-old man who had a history of AMI. The patient had an implantable cardioverter-defibrillator (ICD) for burst ventricular tachycardia. He died suddenly 2 years after ICD implantation. The other patient was a 46-year-old man who had AMI 2.3 months prior to hospitalization. He died suddenly 3 weeks after hospital discharge. The remaining patients had no heart failure symptoms and were classified as NYHA I–II.

**Figure 2:**
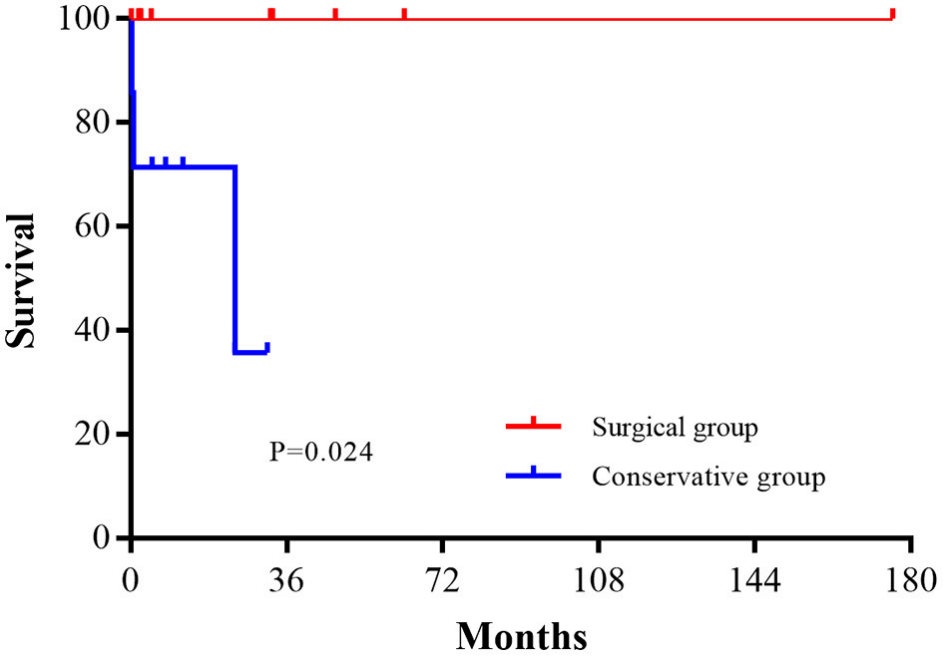
Survival after surgical and conservative treatment.

The estimated survival rate in the conservative group was 36% at 24 months. There was a significant difference in survival between the surgical and conservative groups (*P* = 0.024), as shown in Figure 2.

## Discussion

The major finding of our study is that surgical repair of post-infarction LV pseudoaneurysm can be performed with satisfactory results, while conservative treatment shows poor outcomes.

### Surgery Outcomes

Our study showed that surgical repair of post-infarction pseudoaneurysm yields satisfactory results, with or without concomitant CABG. In our study, there was no in-hospital or follow-up mortality, and all surgically treated patients had good functional status during a follow-up period of 18.5 months. The pre-discharge echocardiographic results also improved after surgery. A review by Frances et al. reported a mortality rate of 23% from 1966 to 1997 (8). In the Mayo Clinic, the surgical mortality rate was 7% (10). The Cleveland Clinic reported a larger group of 30 patients with a 20% hospital death rate, and major morbidities included tracheostomy (27%), dialysis (13%), and re-exploration for bleeding (20%) (6). Studies in Switzerland (11) and Turkey (12) reported an operative mortality rate of about 30%. Surgical treatment is complex and should be performed by experienced surgeons. IABP implantation was required in two cases (20%). In addition, concomitant CABG was indicated to avoid further ischemic cardiomyopathy. Complete revascularization was attempted for every patient to ensure long-term outcomes.

In our study, the conservative group had poor survival (36% over 2 years) which corresponded to high mortality (48%) reported in medically treated patients in other studies (8, 10). Propensity for fatal rupture, or risk of sudden death, seemed high in all medically treated patients, even if they survived the acute phase of AMI and were asymptomatic.

### Indication of Surgery

Pseudoaneurysms are rare and as such their natural history is not well established. They are prone to rupture, leading to sudden death and poor prognosis. When a patient shows symptoms, or when a large pseudoaneurysm is discovered within the first 2 to 3 months after AMI, surgical resection is advisable (13). However, a pseudoaneurysm may be found during echocardiography in otherwise asymptomatic patients that have recovered from AMI, even many years after the AMI. Some are small (<3 cm), with no evidence of expansion and, in these cases, the indication for surgery is controversial (14).

Pretre et al. suggested that chronic asymptomatic pseudoaneurysms <3 cm in diameter and without evidence of expansion might be treated conservatively (11). Frances et al. reported that there is a high mortality rate, regardless of treatment. They observed prolonged survival, even in a few patients who did not undergo surgery (8). The Mayo Clinic also suggest that conservative management in patients with small chronic pseudoaneurysms is reasonable (10). On the other hand, Atik et al. at the Cleveland Clinic insist that surgery should be indicated for everyone diagnosed with LV pseudoaneurysm (6).

In our study, patients chose conservative treatment mainly because they showed no symptoms. However, this group of conservatively treated patients had a poor outcome. There were three (42.9%) deaths, all of which were cardiac-related. The mode of death is sudden death, probably caused by the rupture of the pseudoaneurysm or arrhythmia. Thus, we believe that surgery should be indicated for everyone, regardless of symptoms, AMI interval, and size of the pseudoaneurysm, unless there is a prohibitive risk.

### Timing of Surgery

With the increasing application of echocardiography after AMI, the timing of surgery might be altered. In the Cleveland Clinic, LV pseudoaneurysm was diagnosed a median of 50 days after AMI (10). In our study, the median interval between AMI and hospitalization was 5.7 months. In the surgical group, two (20%) patients underwent surgery within 1 month after AMI and four (40%) within 3 months after AMI. Due to the limited number of cases, we did not analyze the outcomes at different time intervals after AMI.

Early surgery is associated with increased operative mortality (11, 15), which should be considered along with the risk of rupture and hemodynamic deterioration.

Another concern is residual/recurrent communication in the pseudoaneurysm neck (16). In our study, there was one case (10%) of a 1-mm residual communication at pre-discharge TTE but was not found at follow-up. Atik et al. found no residual communication but one case of recurrent communication due to infection (6). Usually, 4 weeks after MI, mature hyalinized fibrous tissue that can be used to anchor sutures is formed, and primary repair is warranted (17). Although delayed surgery might allow secure repair and prevent residual/recurrent communication, it also carries a substantial risk of sudden rupture. It is important to carefully define the suture line intraoperatively, as progressive necrosis of the myocardium near the suture line leads to dehiscence (18). In post-infarction LV pseudoaneurysms, there is usually a small neck, and the surrounding pericardium can be left adherent as reinforcement. Thus, risk of residual communication should not be a concern to delay the surgery.

The precise timing of surgery represents a balance between the risk of surgery and sudden rupture. If the patient is hemodynamically stable, we believe that surgery could be performed after 2–4 weeks of optimized medical treatment.

### Surgery Technique

Occasionally, it is necessary to begin CPB through the femoral artery and vein during deep hypothermal circulatory arrest (19). At the Cleveland clinic, approximately 13% of patients underwent CPB via femoral vessels, and the femoral vessels were routinely exposed during redo surgery (6). We performed arterial cannulation through the ascending aorta in all cases without rupture of the pseudoaneurysm. Severe pericardial adhesions were always observed, which prevented sudden hemorrhage during entry. When the pericardium is opened, cannulation, and clamping should be performed rapidly to avoid rupture or systemic embolization.

Repair of an LV pseudoaneurysm is most commonly performed through median sternotomy and ventriculotomy, but LV pseudoaneurysm repair through left thoracotomy (20), right thoracotomy, and even endoscopic repair have been reported in isolated cases (21). There have been cases in which the trans-mitral endocavitary approach was employed (22) that is particularly useful for patients undergoing concomitant mitral valve surgery. With this approach, exposure, and repair of the pseudoaneurysm neck is achieved through the mitral valve. For all patients, we used the trans-epicardial instead of the transmitral approach. Using this approach, sutures appear to be more precise and definite. The capsule of the pseudoaneurysm can be used as a second layer to cover the repaired pseudoaneurysm neck for reinforcement and the redundant capsule can be resected. In addition, surgeons are more familiar with this approach, as it is similar to the technique used for true aneurysms.

As in the Cleveland Clinic (6), both direct linear closure (*n* = 6, 60%) and patch plasty (*n* = 4, 40%) were used in our institute. Direct linear closure was selected in patients with a relatively small pseudoaneurysm neck and densely fibrotic edges.

Concomitant mitral valve repair in this group of patients should be done using a simply method (23). If there is doubt regarding repair durability, mitral valve replacement should be selected without hesitation.

Percutaneous repair of an LV pseudoaneurysm using a retrograde approach across the aortic valve has been previously described (24), but as the long-term result of percutaneous repair is not well documented, surgical repair is the first choice in our institute.

## Limitations

This was a single-institution clinical cohort study involving a small number of patients. Our institution is a comprehensive heart center, and the first diagnosis is often made in local hospitals or primary heart centers. Only patients referred to our institution were included in this study, which may have introduced a selection bias. Additionally, the patients chose to receive surgical or conservative treatment and conservative treatment was selected because the patient showed no symptoms. It was difficult to persuade them to undergo surgery, especially when the surgery is challenging and the results remain uncertain.

In conclusion, the major finding of our study is that surgical repair of post-infarction pseudoaneurysms can be performed with satisfactory results. Watchful monitoring is associated with a risk of rupture, thus, surgical repair is indicated for every patient diagnosed, even for those with a small pseudoaneurysm without symptoms.

## Data Availability Statement

The original contributions presented in the study are included in the article/supplementary material, further inquiries can be directed to the corresponding author.

## Ethics Statement

The studies involving human participants were reviewed and approved by Institutional Review Board of Fuwai Hospital. The patients/participants provided their written informed consent to participate in this study.

## Author Contributions

ZZ and SL conceived and designed the study. ZZ collected and analyzed the data and wrote the manuscript. ZZ, WS, SZ, and SL reviewed and edited the manuscript. All authors contributed to the manuscript and approved the submitted version.

## Funding

This study was funded by the Capital Science and Technology Program, Beijing (Grant number: Z201100005520005).

## Conflict of Interest

The authors declare that the research was conducted in the absence of any commercial or financial relationships that could be construed as a potential conflict of interest.

## Publisher's Note

All claims expressed in this article are solely those of the authors and do not necessarily represent those of their affiliated organizations, or those of the publisher, the editors and the reviewers. Any product that may be evaluated in this article, or claim that may be made by its manufacturer, is not guaranteed or endorsed by the publisher.
